# Bis[(1*S*,1′*S*)-1,1′-(4-amino-4*H*-1,2,4-triazole-3,5-di­yl)diethanol-κ*N* ^1^]bis­(nitrato-κ*O*)zinc

**DOI:** 10.1107/S1600536812001754

**Published:** 2012-01-21

**Authors:** Xun-Gao Liu, Liang Shen, Qi-Suo Cai, Ma Luo

**Affiliations:** a College of Material Chemistry and Chemical Engineering, Hangzhou Normal University, Hangzhou 310036, People’s Republic of China; b State Key Laboratory of Coordination Chemistry, Coordination Chemistry Institute, School of Chemistry and Chemical Engineering, Nanjing University, Nanjing 210093, People’s Republic of China

## Abstract

In the title homochiral mononuclear compound, [Zn(NO_3_)_2_(C_6_H_12_N_4_O_2_)_2_], the Zn^II^ atom is located on a twofold rotation axis and coordinated by two N atoms from two ligands and two O atoms from two NO_3_^−^ anions, adopting a distorted tetra­hedral coordination geometry. The compound is enanti­omerically pure and corresponds to the *S* diastereoisomer, with the optical activity originating from the chiral ligand. In the crystal, mol­ecules are connected into three-dimensional supra­molecular networks through O—H⋯O, O—H⋯N and N—H⋯O hydrogen bonds.

## Related literature

For 4-amino-4*H*-1,2,4-triazole transition metal complexes, see: Zhai *et al.* (2006 ▶); Yi *et al.* (2004 ▶). For the non-linear optical properties of chiral coordination compounds, see: Evans & Lin (2002 ▶). For uses of chiral coordination compounds, see: Hang *et al.* (2011 ▶); Lin (2010 ▶).
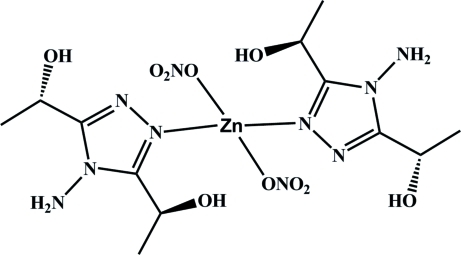


## Experimental

### 

#### Crystal data

[Zn(NO_3_)_2_(C_6_H_12_N_4_O_2_)_2_]
*M* *_r_* = 533.78
Tetragonal, 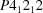

*a* = 12.1252 (7) Å
*c* = 14.6108 (17) Å
*V* = 2148.1 (3) Å^3^
*Z* = 4
Mo *K*α radiation
μ = 1.22 mm^−1^
*T* = 298 K
0.36 × 0.18 × 0.12 mm

#### Data collection

Bruker APEX DUO diffractometer
Absorption correction: multi-scan (*SADABS*; Bruker, 2000 ▶) *T*_min_ = 0.767, *T*_max_ = 0.862
7446 measured reflections
2463 independent reflections
2154 reflections with *I* > 2σ(*I*)
*R*_int_ = 0.032

#### Refinement

*R*[*F* ^2^ > 2σ(*F* ^2^)] = 0.033
*wR*(*F* ^2^) = 0.088
*S* = 1.05
2463 reflections
154 parameters
378 restraints
H-atom parameters constrained
Δρ_max_ = 0.32 e Å^−3^
Δρ_min_ = −0.23 e Å^−3^
Absolute structure: Flack (1983 ▶), 993 Friedel pairs
Flack parameter: −0.022 (15)

### 

Data collection: *SMART* (Bruker, 2000 ▶); cell refinement: *SAINT-Plus* (Bruker, 2000 ▶); data reduction: *SAINT-Plus*; program(s) used to solve structure: *SHELXTL* (Sheldrick, 2008 ▶); program(s) used to refine structure: *SHELXTL*; molecular graphics: *SHELXTL* and *DIAMOND* (Brandenburg & Putz, 2007 ▶); software used to prepare material for publication: *SHELXTL*.

## Supplementary Material

Crystal structure: contains datablock(s) I, global. DOI: 10.1107/S1600536812001754/ff2051sup1.cif

Structure factors: contains datablock(s) I. DOI: 10.1107/S1600536812001754/ff2051Isup2.hkl

Additional supplementary materials:  crystallographic information; 3D view; checkCIF report

## Figures and Tables

**Table 1:** Selected geometric parameters (Å, °)

Zn1—N1	2.0239 (19)
Zn1—O3	2.071 (2)

**Table 2:** Hydrogen-bond geometry (Å, °)

*D*—H⋯*A*	*D*—H	H⋯*A*	*D*⋯*A*	*D*—H⋯*A*
O1—H1⋯O2^ii^	0.82	2.07	2.867 (3)	163
N4—H4*B*⋯O5^ii^	0.89	2.51	3.142 (5)	129
O2—H2⋯N2^iii^	0.82	2.13	2.943 (3)	174
N4—H4*C*⋯O4^iv^	0.89	2.40	3.022 (4)	127

Symmetry codes: (ii) 
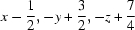
; (iii) 
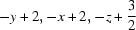
; (iv) 
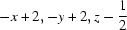
.

**Fig. 1.**
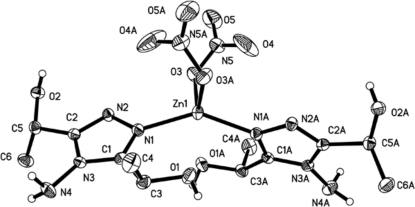
Coordination geometry of Znic in the title compound with atomic labeling scheme. Thermal ellipsoids are at the 30% probability level. All H atoms except those attached to O and N atoms are omitted for clarity.

**Fig. 2.**
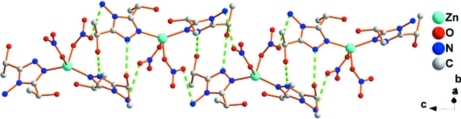
The hydrogen-bonded chains in the title compound along the c axis.

**Fig. 3.**
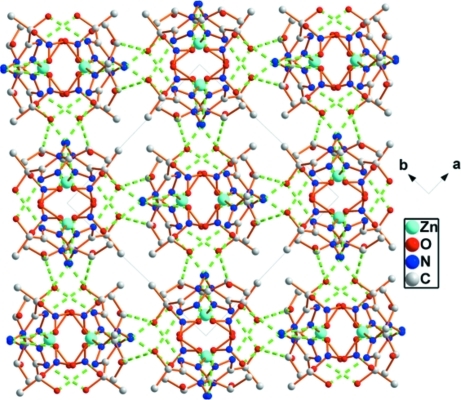
Three dimensional hydrogen-bonded supramolecular networks in the title compound Viewed from c dimension.

## supplementary crystallographic information

### Comment

Chiral coordination complexes have received considerable attention due to their potential applications in the area of ferroelectrics, enantiopure catalysis and separation (Hang *et al.*, 2011; Lin *et al.*, 2010). Among the different approaches to synthesize chiral coordination compounds, the most effective synthetic strategy is to use optically pure chiral ligands. Herein, we report a chiral zinc coordination compound (S)-[Zn(deoatrz)_2_(NO_3_)_2_], by using an enantiopure (1*S*,1'*S*)-1,1'-(4-amino-4*H*-1,2,4-triazole-3,5-diyl)diethanol (deoatrz), reacting with zinc salts. Furthermore, its structure is characterized.

Single crystal structural analysis reveals that the title compound crystallizes in the tetragonal system, chiral space group P4_1_2_1_2. The title compound is a mononuclear and its asymmetric unit consists of one Zn atom, two deoatrz ligands and two NO_3_– anions (Fig. 1). The Zn atom is coordinated by two N atoms (N1, N1A) from two deoatrz ligands and two NO_3_– anions (O3, O3A), adopting a distorted tetrahedral coordination geometry. The Zn—O and Zn—N bond lengths are 2.071 (2) and 2.024 (1) Å, respectively. The bond angles around Zn atom vary from 95.9 (8) to 143.5 (1)*^o^*.

As shown in Fig. 2, the interesting H-bonds are observed in the title compound. The fundamentally units are one dimensional chiral hydrogen bond chains along *c* axis, and subsequently the chains are connected into three-dimensional chiral supramolecular networks through O—H···O, O—H···N and N—H···O hydrogen bond interactions.

### Experimental

An 10 ml ethanol solution of (1*S*,1'*S*)-1,1'-(4-amino-4*H*-1,2,4-triazole -3,5-diyl)diethanol (0.2 mmol, 0.0344 g) and Zn(NO_3_)_2_._6_H_2_O (0.10 mmol, 0.0298 g) was stirred for five minutes and then filtered. The filtrate was carefully layered with 10 ml ethyl ether. After one week, colorless needle-like crystals of the title compound were obtained. Yield: 45%.

### Refinement

All H atoms were put in calculated positions. All H atoms were refined with *U*_iso_(H) = 1.2*U*_eq_(N and O) and *U*_iso_(H) = 1.2 or 1.5*U*_eq_(C).

### Crystal data

**Table:**

[Zn(NO_3_)_2_(C_6_H_12_N_4_O_2_)_2_]	*D*_x_ = 1.651 Mg m^−^^3^
*M**_r_* = 533.78	Mo *K*α radiation, λ = 0.71073 Å
Tetragonal, *P*4_1_2_1_2	Cell parameters from 7446 reflections
Hall symbol: P 4abw 2nw	θ = 1.7–27.5°
*a* = 12.1252 (7) Å	µ = 1.22 mm^−^^1^
*c* = 14.6108 (17) Å	*T* = 298 K
*V* = 2148.1 (3) Å^3^	Needle, colourless
*Z* = 4	0.36 × 0.18 × 0.12 mm
*F*(000) = 1104	

### Data collection

**Table:**

Bruker APEX DUO diffractometer	2463 independent reflections
Radiation source: fine-focus sealed tube	2154 reflections with *I* > 2σ(*I*)
graphite	*R*_int_ = 0.032
φ and ω scans	θ_max_ = 27.5°, θ_min_ = 2.4°
Absorption correction: multi-scan (*SADABS*; Bruker, 2000)	*h* = −15→9
*T*_min_ = 0.767, *T*_max_ = 0.862	*k* = −15→13
7446 measured reflections	*l* = −18→12

### Refinement

**Table:**

Refinement on *F*^2^	Secondary atom site location: difference Fourier map
Least-squares matrix: full	Hydrogen site location: inferred from neighbouring sites
*R*[*F*^2^ > 2σ(*F*^2^)] = 0.033	H-atom parameters constrained
*wR*(*F*^2^) = 0.088	*w* = 1/[σ^2^(*F*_o_^2^) + (0.0455*P*)^2^ + 0.2512*P*] where *P* = (*F*_o_^2^ + 2*F*_c_^2^)/3
*S* = 1.05	(Δ/σ)_max_ = 0.001
2463 reflections	Δρ_max_ = 0.32 e Å^−^^3^
154 parameters	Δρ_min_ = −0.23 e Å^−^^3^
378 restraints	Absolute structure: Flack (1983), 993 Friedel pairs
Primary atom site location: structure-invariant direct methods	Flack parameter: −0.022 (15)

### Special details

**Table:**

Geometry. All e.s.d.'s (except the e.s.d. in the dihedral angle between two l.s. planes) are estimated using the full covariance matrix. The cell e.s.d.'s are taken into account individually in the estimation of e.s.d.'s in distances, angles and torsion angles; correlations between e.s.d.'s in cell parameters are only used when they are defined by crystal symmetry. An approximate (isotropic) treatment of cell e.s.d.'s is used for estimating e.s.d.'s involving l.s. planes.
Refinement. Refinement of *F*^2^ against ALL reflections. The weighted *R*-factor *wR* and goodness of fit *S* are based on *F*^2^, conventional *R*-factors *R* are based on *F*, with *F* set to zero for negative *F*^2^. The threshold expression of *F*^2^ > σ(*F*^2^) is used only for calculating *R*-factors(gt) *etc*. and is not relevant to the choice of reflections for refinement. *R*-factors based on *F*^2^ are statistically about twice as large as those based on *F*, and *R*- factors based on ALL data will be even larger.

### Fractional atomic coordinates and isotropic or equivalent isotropic displacement parameters (Å^2^)

**Table:**

	*x*	*y*	*z*	*U*_iso_*/*U*_eq_	
Zn1	0.92340 (3)	0.92340 (3)	1.0000	0.03222 (13)	
O1	0.72436 (18)	0.90461 (18)	0.95521 (12)	0.0417 (5)	
H1	0.7021	0.8493	0.9817	0.063*	
O2	1.13628 (15)	0.75723 (17)	0.67187 (13)	0.0336 (4)	
H2	1.1479	0.8235	0.6777	0.050*	
O3	1.09002 (17)	0.89583 (19)	1.02136 (13)	0.0464 (5)	
O4	1.0802 (3)	1.0273 (3)	1.1173 (3)	0.1176 (14)	
O5	1.2371 (2)	0.9742 (3)	1.0727 (2)	0.0758 (9)	
N1	0.91797 (19)	0.85483 (18)	0.87379 (13)	0.0285 (5)	
N2	1.00505 (19)	0.81575 (19)	0.82025 (13)	0.0279 (5)	
N3	0.84957 (17)	0.80180 (17)	0.74473 (15)	0.0271 (4)	
N4	0.7738 (2)	0.7823 (2)	0.67330 (16)	0.0427 (6)	
H4B	0.7928	0.7208	0.6440	0.064*	
H4C	0.7753	0.8388	0.6345	0.064*	
N5	1.1372 (2)	0.9678 (2)	1.07029 (17)	0.0417 (6)	
C1	0.8266 (2)	0.8474 (2)	0.82697 (16)	0.0273 (5)	
C2	0.9608 (2)	0.7841 (2)	0.74215 (16)	0.0262 (5)	
C3	0.7171 (2)	0.8883 (2)	0.85876 (17)	0.0332 (6)	
H3	0.6595	0.8343	0.8446	0.040*	
C4	0.6903 (3)	0.9988 (3)	0.8141 (2)	0.0467 (8)	
H4D	0.6216	1.0261	0.8377	0.070*	
H4E	0.6844	0.9892	0.7490	0.070*	
H4F	0.7479	1.0507	0.8274	0.070*	
C5	1.0219 (2)	0.7390 (2)	0.66148 (17)	0.0299 (6)	
H5	0.9968	0.7776	0.6063	0.036*	
C6	1.0044 (3)	0.6164 (3)	0.6482 (2)	0.0460 (8)	
H6A	1.0389	0.5934	0.5923	0.069*	
H6B	0.9268	0.6011	0.6452	0.069*	
H6C	1.0363	0.5770	0.6987	0.069*	

### Atomic displacement parameters (Å^2^)

**Table:**

	*U*^11^	*U*^22^	*U*^33^	*U*^12^	*U*^13^	*U*^23^
Zn1	0.03548 (17)	0.03548 (17)	0.02569 (18)	0.0024 (2)	0.00483 (13)	−0.00483 (13)
O1	0.0449 (12)	0.0468 (14)	0.0334 (9)	−0.0032 (10)	0.0117 (9)	0.0021 (9)
O2	0.0277 (10)	0.0310 (11)	0.0420 (9)	0.0005 (8)	0.0038 (8)	−0.0056 (9)
O3	0.0380 (12)	0.0514 (13)	0.0497 (10)	0.0025 (9)	−0.0045 (9)	−0.0172 (9)
O4	0.0629 (19)	0.112 (3)	0.178 (3)	0.0013 (19)	−0.005 (2)	−0.105 (3)
O5	0.0356 (15)	0.102 (2)	0.0901 (18)	0.0029 (14)	−0.0133 (13)	−0.0283 (17)
N1	0.0269 (11)	0.0310 (11)	0.0277 (9)	0.0003 (10)	0.0016 (9)	−0.0025 (8)
N2	0.0260 (11)	0.0307 (11)	0.0269 (9)	0.0038 (9)	−0.0002 (8)	−0.0031 (8)
N3	0.0242 (10)	0.0296 (10)	0.0275 (8)	−0.0036 (8)	−0.0028 (9)	−0.0012 (9)
N4	0.0409 (14)	0.0495 (16)	0.0378 (10)	−0.0041 (11)	−0.0161 (11)	−0.0046 (12)
N5	0.0369 (15)	0.0375 (14)	0.0508 (13)	0.0048 (11)	−0.0053 (11)	−0.0062 (12)
C1	0.0282 (13)	0.0250 (13)	0.0288 (10)	−0.0015 (10)	0.0020 (10)	0.0035 (10)
C2	0.0265 (11)	0.0248 (12)	0.0272 (10)	−0.0010 (9)	−0.0026 (10)	0.0020 (10)
C3	0.0265 (14)	0.0394 (15)	0.0336 (11)	−0.0004 (11)	0.0022 (10)	0.0016 (11)
C4	0.0406 (18)	0.0481 (19)	0.0515 (16)	0.0162 (15)	0.0033 (14)	0.0044 (15)
C5	0.0278 (13)	0.0339 (14)	0.0281 (10)	0.0028 (11)	−0.0015 (10)	−0.0028 (10)
C6	0.0423 (18)	0.0410 (18)	0.0546 (17)	−0.0048 (15)	0.0031 (15)	−0.0171 (14)

### Geometric parameters (Å, °)

**Table:**

Zn1—N1	2.0239 (19)	N3—N4	1.410 (3)
Zn1—N1^i^	2.0239 (19)	N4—H4B	0.8900
Zn1—O3	2.071 (2)	N4—H4C	0.8900
Zn1—O3^i^	2.071 (2)	C1—C3	1.492 (4)
O1—C3	1.426 (3)	C2—C5	1.496 (4)
O1—H1	0.8200	C3—C4	1.525 (4)
O2—C5	1.412 (3)	C3—H3	0.9800
O2—H2	0.8200	C4—H4D	0.9600
O3—N5	1.265 (3)	C4—H4E	0.9600
O4—N5	1.212 (4)	C4—H4F	0.9600
O5—N5	1.215 (4)	C5—C6	1.514 (4)
N1—C1	1.305 (3)	C5—H5	0.9800
N1—N2	1.397 (3)	C6—H6A	0.9600
N2—C2	1.318 (3)	C6—H6B	0.9600
N3—C1	1.352 (3)	C6—H6C	0.9600
N3—C2	1.366 (3)		
			
N1—Zn1—N1^i^	143.46 (13)	N2—C2—C5	125.9 (2)
N1—Zn1—O3	95.90 (8)	N3—C2—C5	124.6 (2)
N1^i^—Zn1—O3	104.96 (8)	O1—C3—C1	107.4 (2)
N1—Zn1—O3^i^	104.96 (8)	O1—C3—C4	108.4 (2)
N1^i^—Zn1—O3^i^	95.90 (8)	C1—C3—C4	110.4 (2)
O3—Zn1—O3^i^	109.73 (13)	O1—C3—H3	110.2
C3—O1—H1	109.5	C1—C3—H3	110.2
C5—O2—H2	109.5	C4—C3—H3	110.2
N5—O3—Zn1	114.52 (17)	C3—C4—H4D	109.5
C1—N1—N2	108.94 (19)	C3—C4—H4E	109.5
C1—N1—Zn1	122.26 (18)	H4D—C4—H4E	109.5
N2—N1—Zn1	128.68 (16)	C3—C4—H4F	109.5
C2—N2—N1	106.0 (2)	H4D—C4—H4F	109.5
C1—N3—C2	107.0 (2)	H4E—C4—H4F	109.5
C1—N3—N4	126.3 (2)	O2—C5—C2	110.2 (2)
C2—N3—N4	126.6 (2)	O2—C5—C6	107.8 (2)
N3—N4—H4B	109.2	C2—C5—C6	113.0 (2)
N3—N4—H4C	109.2	O2—C5—H5	108.6
H4B—N4—H4C	109.5	C2—C5—H5	108.6
O4—N5—O5	120.9 (3)	C6—C5—H5	108.6
O4—N5—O3	118.2 (3)	C5—C6—H6A	109.5
O5—N5—O3	120.8 (3)	C5—C6—H6B	109.5
N1—C1—N3	108.6 (2)	H6A—C6—H6B	109.5
N1—C1—C3	124.7 (2)	C5—C6—H6C	109.5
N3—C1—C3	126.6 (2)	H6A—C6—H6C	109.5
N2—C2—N3	109.4 (2)	H6B—C6—H6C	109.5

Symmetry codes: (i) *y*, *x*, −*z*+2.

### Hydrogen-bond geometry (Å, °)

**Table:**

*D*—H···*A*	*D*—H	H···*A*	*D*···*A*	*D*—H···*A*
O1—H1···O2^ii^	0.82	2.07	2.867 (3)	163
N4—H4B···O5^ii^	0.89	2.51	3.142 (5)	129
O2—H2···N2^iii^	0.82	2.13	2.943 (3)	174
N4—H4C···O4^iv^	0.89	2.40	3.022 (4)	127

Symmetry codes: (ii) *x*−1/2, −*y*+3/2, −*z*+7/4; (iii) −*y*+2, −*x*+2, −*z*+3/2; (iv) −*x*+2, −*y*+2, *z*−1/2.

## References

[bb1] Brandenburg, K. & Putz, H. (2007). *DIAMOND* Crystal Impact GbR, Bonn, Germany.

[bb2] Bruker (2000). *SMART*, *SADABS* and *SAINT-Plus* Bruker AXS Inc., Madison, Wisconsin, USA.

[bb3] Evans, O. R. & Lin, W. (2002). *Acc. Chem. Res.* **35**, 511–522. 10.1021/ar0001012 12118990

[bb4] Flack, H. D. (1983). *Acta Cryst.* A**39**, 876–881.

[bb5] Hang, T., Zhang, W., Ye, H.-Y. & Xiong, R.-G. (2011). *Chem. Soc. Rev.* **40**, 3577–3598. 10.1039/c0cs00226g 21509354

[bb6] Lin, W. (2010). *Top. Catal.* **53**, 869–875.

[bb7] Sheldrick, G. M. (2008). *Acta Cryst.* A**64**, 112–122. 10.1107/S0108767307043930 18156677

[bb8] Yi, L., Ding, B., Zhao, B., Cheng, P., Liao, D.-Z., Yan, S.-P. & Jiang, Z.-H. (2004). *Inorg. Chem.* **43**, 33–43. 10.1021/ic0348244 14704051

[bb9] Zhai, Q.-G., Wu, X.-Y., Chen, S.-M., Lu, C.-Z. & Yang, W.-B. (2006). *Cryst. Growth Des.* **6**, 2126–2135.

